# A Standardized Protocol for Mouse Longevity Studies in Preclinical Drug Development

**DOI:** 10.14336/AD.2025.0508

**Published:** 2026-06-02

**Authors:** Alex Zhavoronkov, Qian Wang, Yujie Liu, Wenbin Hou, Yuelei Shen, Dominika Wilczok, Kristen Fortney, Alex Aliper, Man Zhang, Feng Ren, Richard A. Miller

**Affiliations:** ^1^Insilico Medicine US Inc., Cambridge, MA 02138, USA.; ^2^Insilico Medicine AI Limited, Masdar, Abu Dhabi, United Arab Emirates.; ^3^Insilico Medicine Hong Kong Ltd., Hong Kong SAR, China.; ^4^Insilico Medicine Shanghai Ltd., Shanghai 201203, China.; ^5^Biocytogen Pharmaceuticals (Beijing) Co., Ltd., Beijing 102609, China.; ^6^Duke University, Durham, NC 27708, USA.; ^7^Duke Kunshan University, Kunshan 215316, Jiangsu, China.; ^8^BioAge Labs, Richmond, CA 94804, USA.; ^9^Department of Pathology and Geriatrics Center, University of Michigan, Ann Arbor, MI 48109-5796, USA.

**Keywords:** Aging, Lifespan Studies, Preclinical Drug Development, Mouse Models, Chronic Disease Therapeutics, Geroprotective Interventions

## Abstract

Although aging is increasingly recognized as a key factor in chronic disease management, preclinical drug development rarely incorporates direct assessments of lifespan. To date, no biotechnology company has conducted a full mouse lifespan study for a therapeutic agent prior to human clinical trials, despite widespread chronic use of many approved drugs. This oversight stems from a lack of standardized protocols for the incorporation of mouse lifespan studies, high costs, limited commercial incentives, and regulatory risks associated with long-term data. Here, we present a comprehensive and scalable protocol for conducting mouse longevity studies in the early stages of drug development. Being aware of monetary constraints in the drug discovery process, we propose a basic design for a longevity study on ~250 (176 males and 72 females) genetically heterogeneous mice (UM-HET3) per group, with survival curves as primary endpoint, and propose enhanced study design options only if budget allows. Our framework provides a standardized foundation for integrating longevity assessments into routine drug development, offering the potential to uncover long-term risks or benefits that traditional toxicology studies may overlook. Broad implementation of such protocols could support the development of safer and more effective therapeutics for chronic diseases, while opening new avenues for discovery of substances that could slow down the rate of aging, known as geroprotectors.

## Introduction

Since the advent of modern targeted drug discovery (2004-2024), 719 drugs (vaccine and gene therapy not included) have been approved by FDA, of which 549 are new molecular entities (NME) and 170 are biologics. In 2023, 53 out of 55 approved drugs were developed for novel targets [1]. Drug repurposing, with success in a dozen cases, still faces large challenges [2].

Aside from a few cases where aging research was used as a platform for drug discovery in longevity biotechnology companies [3], none of which completed all phases of clinical trials, there are no known examples where a biotechnology company performed a lifespan study for their drug in mice or other mammals before entering human clinical trials. However, hundreds of targeted drugs were developed for chronic diseases and intended to be taken for multiple years or the entire life of the patient. These include anti-obesity drugs such as GLP-1 inhibitors (Ozempic, Wegovy, Victoza), erectile dysfunction medications such as PDE5 inhibitors (Viagra, Cialis, Levitra), anti-cholesterol medications such as PCSK9 inhibitors, and many other drugs that target chronic conditions, each of which may have increased risks of side-effects if used for a prolonged time. The biotechnology and pharmaceutical companies in charge of discovery or development of these drugs do not conduct lifespan studies in mammals to evaluate the effects of long-term exposure by replicating human administration of the drugs in mice.

We have identified four main reasons why such studies are not routinely performed:

### 1. Lack of standardized protocols for incorporating mouse lifespan studies into the early drug development stage and contract research organizations (CROs) ready to run such studies in aged mice

While the pharmaceutical industry has well-established workflows for safety and efficacy testing in Investigational New Drug (IND)-enabling studies, standardized protocols for lifespan assessments are not yet integrated into early-stage drug development. Specifically, consensus guidelines on mouse strain selection, sample sizes, intervention timing, endpoints like survival curves, and longitudinal biomarker collection are lacking, hindering reproducibility and scalability. This gap is further compounded by the limited number of CROs equipped to maintain large aging mouse colonies and conduct robust lifespan studies. For these studies to be feasible as part of routine drug development, CROs would need significant infrastructure, including aging mouse colonies and expertise in collecting comprehensive longitudinal biomarker data alongside survival outcomes. However, the current demand for such services is low, leading most CROs to offer customized, resource-intensive, and often limited lifespan study options. Only a limited number of CROs maintain the infrastructure (large aging colonies, gerontology expertise) needed to run full lifespan trials. As a result, if a drug developer commissions a study, the design is tailored to their needs and the CRO’s capabilities. This leads to variability: one CRO might use a certain mouse strain, diet, and monitoring schedule, while another uses different parameters. Most CROs currently offer such studies as a special, resource-intensive service rather than a routine product. Furthermore, CROs typically run these studies at a single site with their own husbandry conditions, so results could be influenced by lab-specific factors (microbiome, caging, etc.) without the cross-validation that multi-site designs provide. The lack of standardization means outcomes from one CRO’s study may not be directly comparable to another’s. To incentivize CROs to provide scalable, standardized services, the demand for lifespan studies must increase, which would require setting lifespan evaluation as an integral part of preclinical drug testing.

An existing proxy for such routine lifespan studies is the NIA-funded Interventions Testing Program (mouse ITP) that performs lifespan studies on compounds that hold promises to extend longevity. As of Aug 2024, the ITP has initiated 108 full lifespan studies (www.nia.nih.gov/research/dab/interventions-testing-program-itp/supported-interventions). Of these, 69 involved a single drug at a single dose, 4 involved a combination of agents, and 35 involved multiple doses and/or start ages. Eighty-five lifespan studies (Cohorts 2004 – 2020) are now complete and published in peer-reviewed journals and another 31 (Cohorts 2021 – 2024) are in progress. Since its inception, ITP has completed 85 lifespan studies of which 55 compounds and combinations in lifespan studies, among which 21 were considered as targeted therapeutics, and 19 were tested post-FDA approval. Twelve compounds used as single agent demonstrated significant lifespan extension in mice [4]. Table 1 shows drugs tested in ITP post FDA approval, and their demonstrated effect on lifespan. While the ITP serves as a valuable model for standardized lifespan studies, and the golden standard of mouse longevity studies, its government-funded nature, USA-centric infrastructure, and limited on-demand accessibility make it challenging to integrate into commercial drug development pipelines. Unlike industry-driven CRO services, the ITP operates on a fixed schedule and selection process, limiting its applicability for companies requiring flexibility, rapid turnaround, and tailored study designs aligned with their drug development timelines.

**Table 1.** Drugs Approved by the FDA Between 2004-2024 Tested in ITP Lifespan Studies

Name	FDA approval year	Indication	Lifespan extension
**Fish Oil (e.g. Omacor/Lovaza)**	2004	Hypertriglyceridemia	No
**Nebivolol**	2007	Hypertension	No
**Dimethyl Fumarate**	2013	Multiple sclerosis	No
**Canagliflozin**	2013	Type 2 diabetes	Yes, male

### 2. Increased risk to the preclinical and clinical programs if side effects are noted after long-term treatment of mice

Standard regulatory toxicology studies, spanning weeks to months, effectively detect early organ damage but often miss toxicities that accumulate slowly or emerge in late life. Mouse lifespan studies could serve as a long-term ‘stress test’ for drugs intended for chronic use. These studies, when performed alongside IND phase, would generate survival curves, cause-of-death data, and age-stratified biomarkers that can be included in regulatory submissions as exploratory evidence. Such data could uncover delayed organ-specific toxicities undetected by standard studies, guide enhanced safety monitoring in early-phase trials and validate exposure margins, as well as provide preliminary evidence of geroprotective or healthspan benefits, potentially supporting future label extensions once regulatory pathways are defined. However, regulatory agencies like the FDA or EMA may request further studies to interpret these findings, potentially extending approval timelines and costs. Additionally, late-emerging toxicities that could be identified in lifespan studies could complicate preclinical and clinical development, necessitating careful risk-benefit assessments.

### 3. High cost and long duration of the study

The financial and logistical demands of longevity studies involving mice are considerable, with costs increasing significantly for older animals. As of March 2025, the price of UM-HET3 mice at Jaxon Laboratories varied by age: $77.70 for 16-week-old mice, $133.06 for 24-week-old mice, and $206.37 for 40-week-old mice (https://www.jax.org/strain/036603). Given the cohort information reported by ITP (https://phenome.jax.org/projects/ITP1/animal), a study would require roughly 500 mice for a two-group design to detect a 10% lifespan extension at a power of 80% and 5% type I error. This would lead to an investment of approximately $38,500–$103,000 for mouse procurement alone, depending on the selected starting age (16–40 weeks). This estimate excludes international shipping costs for locations outside the USA and Canada. Beyond the cost of animal acquisition, additional expenses include drug synthesis, formulation of experimental diets, animal husbandry, biomarker assessments, and administrative overhead, all of which contribute to the overall financial burden of conducting longevity research.

### 4. Unclear benefits to the study sponsor or biotechnology company

There are no formal legal requirements or economic incentives to perform such studies, especially at the early stages of drug discovery. Biotechnology companies are developing drugs for specific indications and not for administration to healthy people and are initially uninterested in new indications not supported by preclinical evidence or direct biological insights. Moreover, commercial biotechnology companies and investors face limited financial motivation to develop and conduct expensive clinical studies intended to demonstrate efficacy and long-term safety for generic drugs and drugs with limited patent life. Therefore, most of the drugs tested in the ITP post FDA approval are unlikely to be tested in human clinical trials for longevity. One such example is metformin, for which the human clinical trial called Targeting Aging with Metformin (TAME) was proposed around 2015 [5]. Advocates of the TAME project needed to seek public and non-profit funding, because it is not commercially viable for profit-seeking companies to conduct an expensive and long-term trial for a generic drug.

**Figure 1.**
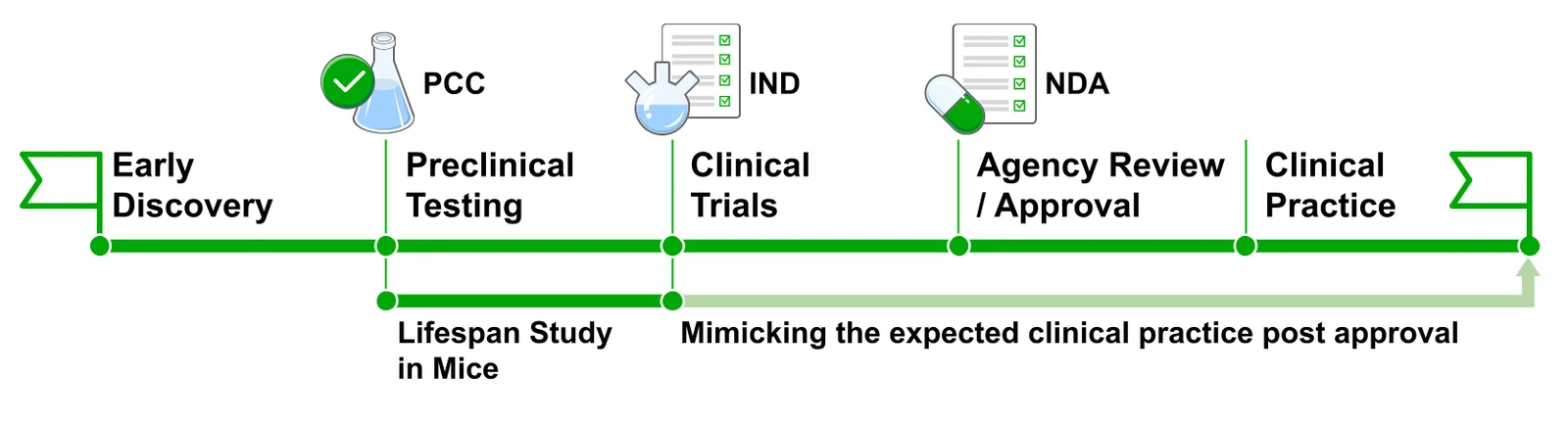
**Position of mouse longevity study as a part of the drug development process**. The diagram illustrates a parallel track in which a standardized lifespan study in genetically heterogeneous mice is conducted during preclinical development. This study aims to mimic the expected clinical use post-approval, providing early insights into long-term safety and potential lifespan effects.

To address these systemic barriers in the biotechnology industry, we propose a protocol for conducting routine mouse lifespan studies in early stages of drugs development. We propose that this protocol should be executed for novel drugs that are intended for long term use, as well as for existing, most used drugs that target human chronic diseases. The mouse trials would attempt to replicate the human therapeutic protocol in terms of dose and frequency. This concept may increase the chances of identifying negative or positive side effects of the drugs intended for long-term use as well as evaluate the effects of these drugs on mouse lifespan. Although this strategy does not claim to resolve all translational challenges (differences in metabolism and pharmacokinetics, genetic and physiological disparities, etc.) it could contribute critical data that can inform subsequent, more targeted investigations. This protocol would provide data for evaluating the long-term effects of drugs intended for chronic administration on overall organismal health, which may not be directly observed in short term GLP-tox studies. We recommend that longevity study could be carried out in parallel to or as an extension of long-term tox studies, especially for compounds designed for the treatment of chronic diseases, as indicated in Figure 1. While we recognize that implementing such studies introduces additional costs for drug developers, these costs are minor relative to the potential benefits of obtaining long-term safety and efficacy data.

## Proposed standardized longevity study protocol

Before detailing the lifespan study protocol, it is important to address that the protocol should follow the best animal welfare practice as outlined by the ITP and comply with the local laws and regulations (https://phenome.jax.org/projects/ITP1/animal) as well as must secure ethical approval from an appropriate review board.

To balance cost efficiency with scientific rigor, we propose a Basic Longevity Study Protocol that incorporates the minimum necessary components to ensure reliable and reproducible results in early-stage drug development longevity studies. We argue that these elements should serve as the standard framework for longevity studies at this stage. We outline enhanced study designs that build upon this basic protocol, offering expanded data collection in scenarios where resources permit. These enhancements enable more precise evaluations, refine conclusions, and facilitate targeted follow-up investigations, ultimately strengthening the translational value of lifespan studies.

## Basic Longevity Study Protocol

The primary goal for longevity study is to examine the extent to which the lifespan has been changed by the tested compound in mice.

### 1. Selection of mouse strain

Mice are commonly used in longevity studies. The median lifespan varies dramatically among mouse strains and sexes, ranging from 251 days in female AKR/J mice to 964 days in female WSB/EiJ mice [6]. C57BL/6J mice are commonly used in lifespan studies [7–10] with a median lifespan estimated around 28-30 months depending on housing/diet conditions [11]. However, nearly all these strains are inbred and selected for many generations to be adapted to laboratory maintenance and usage, e.g, rapid maturation and quick cohort expansion. As a result, these lines may be forced to abandon the natural alleles that delay aging. To support this hypothesis, Miller and colleagues generated three lines of mice from wild trapped progenitors, among which two lines demonstrated significant differences in size, body weight, sexual maturation in females and maximal lifespan compared to a representative inbred stock [12]. These observations raise concern on the selection of appropriate strains for longevity studies to balance the study reproducibility and maximal genetic diversity that allows unbiased investigation of longevity mediators. To solve this problem, the ITP mice, also named WEFET3, are generated by breeding two hybrids, (BALB/cByJ × C57BL/6J) F1 × (C3H/HeJ × DBA/2J) F1, so that all mice are genetically heterogeneous but the genetic variation of the population is reproducible [13]. Since 2004, ITP has generated over 20 cohorts of UM-HET3 mice at three sites to test compound intervention on longevity, among which studies from 16 cohorts have been completed and reported (https://phenome.jax.org/projects/ITP1). These accumulating data allow us to comprehensively track and trace how lifespan changes across cohort years and study sites, as well as the data reproducibility under the same study protocol. In principle, genetically heterogeneous mice are recommended over isogenic lines as certain homogeneous mutations may skew the efficacy of the testing compound. Therefore, the authors endorse UM-HET3 mice as the most reliable model for a longevity study in early drug development. If UM-HET3 or its parental lines are not readily available at the study site, one may consider generating a segregating stock with available strains by 4-way crossing, replicating the ITP cohort generation.

Although UM HET3 mice offer the most reproducible genetically heterogeneous background, the fact that interventions acting on conserved nutrient sensing pathways (e.g., mTOR, AMPK, IIS) reproducibly extend lifespan in multiple inbred strains, companion dogs, and invertebrate models, combined with evidence that sex dimorphic outcomes arise from differential gonadal hormone regulation of metabolism and hepatic xenobiotic gene expression, indicates that the results coming out of such lifespan studies could be generalizable to other models while still necessitating sex stratified analyses

### 2. Age to start intervention and intervention duration

Some key questions about lifespan studies are the time to start intervention and intervention duration. Some interventions start around 16-20 months of age in wild-type C57BL/6J mice, which is roughly equivalent to 60 years of age in humans, to mimic the late-life intervention against the natural aging process [14, 15]. Such interventions usually continue until all the mice have died. Others may start at the age of 4-12 months to represent early or mid-life prevention [16, 17] and would continue for a much longer time. The choice of age to start the intervention and study duration is usually based on the drug’s mechanisms of action, the cost of mice maintenance and the cost of the test compound.

### 3. Sample size calculation

Another key decision is the sample size for both sexes to be tested in the study, because evidence demonstrates a sex-dependent response in longevity studies [16, 18–20]. Studies carried out by ITP have established a 10% change in lifespan, 0.05 type I error and 80% power as an accepted standard for power analysis [20]. Based on the survival data reported by ITP (https://phenome.jax.org/projects/ITP1), we included control groups in 2004 to 2020 cohorts (2008 cohort not available) and excluded data points with status listed as ‘removed’. We summarized the survival stats by sex, cohort year and site (Supplemental Table 1 - 4). ANOVA analysis indicates that male mice from different cohort years (F=4.698, P<0.0001) and sites (F=57.21, P<0.0001) demonstrated statistically significant differences in lifespan while lifespan of female mice was only significantly affected by sites (F=3.849, P=0.0214), but not by cohort years (F=1.449, P=0.116).

**Table 2.** Sample Sizes for Male Mice.

Alpha	%Lifespan extension	Power	N	n1	n2	Mean1	Mean2	SD
**0.05**	10	0.8	318	159	159	766.08	842.69	242.64
**0.05**	15	0.8	142	71	71	766.08	880.99	242.64
**0.05**	20	0.8	82	41	41	766.08	919.30	242.64

Given the results, we estimate the sample size needed to detect 10, 15 and 20% lifespan extension at a single site using UM-HET3 mice based on all data combined (cohort years and sites), data from cohort years with smallest and largest standard deviation and data from each site. For male mice, we could obtain an estimated sample size of 159 per group to detect a 10% lifespan with all data combined (Table 2), ranging from 112 to 232 from the cohort years with smallest to largest variation (2006 vs 2017 cohort, Supplemental Table 5 and 6), and from 130 to 170 among different sites (Supplemental Table 7 - 9). For female mice, we could obtain an estimated sample size of 64 per group to detect a 10% lifespan with all data combined (Table 3), ranging from 51 to 80 from the cohort years with smallest to largest variation (2007 vs 2018 cohort, Supplemental Table 11 and 12), and from 62 to 65 among different sites (Supplemental Table 13 - 15). It turned out that sample size estimation correlates well with SD from each cohort year (Supplemental Table 10 and 16). Notably, as the actual survival will be examined by log-rank test, these Mean-SD based sample sizes are approximations for reference.

To account for fighting, we recommend increasing the number of male mice by at least 10%, as fighting is common and results in the inevitable loss of sample size. Other causes of death include failure to recover from blood sampling and laboratory error, approximately accounting for 4% females and 5% males [6]. Therefore, the sample size to detect a 10% change in lifespan, 0.05 type I error and 80% power for male and female would be at least 176 and 72, respectively

The sample size estimation here only applies to a two-group design. If more than one compound is going to be tested in the same cohort, it is recommended to include more mice into the control group to improve the power. For comparison, ITP uses two times more control mice than the count in any one drug-intervention group.

Including more mice is always ideal for detecting subtle changes in lifespan with sufficient statistical power. However, to balance the cost considerations, we recommend taking the following factors into account:

1. Any preliminary findings suggest that the testing compound has a potentially strong impact on lifespan
2. Any preliminary findings indicating a strong sex-specific mode of action

In case 1, if prior data indicate a significant lifespan extension (e.g., ~15%), the study can be designed accordingly by adjusting the expected effect size, while maintaining other parameters the same. This would require approximately 29 females and 71 males per group (see Supplementary Tables 2.1 and 3.1), with additional males to compensate for attrition due to fighting, totaling around 120 mice per group (85 males and 35 females).

In case 2, if there is strong evidence that the intervention is effective only in one sex, a single-sex study design may be considered to reduce the number of animals and cost. However, it is important to note that this approach trades reduced sample size for increased risk of missing an effect due to underpowering or incorrect assumptions about sex-specificity.

**Table 3.** Sample Sizes for Female Mice.

Alpha	%Lifespan extension	Power	N	n1	n2	Mean1	Mean2	SD
0.05	10	0.8	128	64	64	875.08	962.59	174.61
0.05	15	0.8	58	29	29	875.08	1006.34	174.61
0.05	20	0.8	34	17	17	875.08	1050.10	174.61

### 4. Dosing regimen and administration route

The dosing regimen could significantly affect the outcome of the longevity study. With aging, the body's metabolic capacity of drugs declines, and the optimal dosing and frequency should be determined prior in aged mice, e.g. through small scale pilot PK/PD studies, and not simply implied from historical data from young mice during the drug development process. As longevity is different from the original indication the compound has been designed for, whether the dosing regimen that covers the EC50/90 of the original indication will fall into the purpose of lifespan study remains to be investigated.

Moreover, given the prolonged duration of intervention, attention should be paid to the proper route of administration for the sake of animal welfare. For instance, excessive injections can lead to local inflammation and fibrosis, and repetitive gavage may cause damage to the gastrointestinal tract. Therefore, oral administration of testing compounds with high oral bioavailability that can be incorporated into drinking water or diet is a viable approach [21] and is required in ITP studies [14, 17–19]. If a drug is unstable in acidic conditions, formulation encapsulation can be utilized prior to chow diet preparation. For example, encapsulating rapamycin with Eudragit S100 before addition into the chow diet can help reduce the degradation of tested compounds in stomach acid [22]. For testing compounds that cannot be administered orally, such as antibodies with a longer half-life, intravenous injection every 3 weeks is well tolerated in mice [15]. However, such an administration route would require a sham-injected control group with a non-specific antibody of the same species and isotype, which greatly increases the cost. The ideal situation is to include multiple dosing regimens in one longevity study; however, it generates additional costs.

### 5. Basic parameters to be monitored during the study

Many parameters or phenotypes can be monitored during the process of aging but not all of them can predict or reflect adverse health outcomes. In human, frailty phenotype and frailty index (FI) are the two major models describing the age-associated declines in physiological reserve and function across multiorgan systems that lead to increased vulnerability to adverse health outcomes [23]. In aged mice, frailty was initially evaluated by FI using specialist equipment and invasive methods and the results were correlated with those found in humans [24]. A simplified, non-invasive murine FI was further developed to monitor the condition of aged mice and was able to capture a marked increase in FI score in a moribund animal [25], which includes coat/skin, musculoskeletal/physical condition, auditory function, ocular/nasal, digestive/urogenital, respiratory system, discomfort, and temperature and body weight score.

Recent studies suggest that body temperature is a determining factor affecting mouse lifespan [26] and monitoring body temperature alone and temperature and body weight score (T*BW) may serve as a useful predictive index of death/humane endpoint [27]. Therefore, we suggest body weight and body temperature as the basic parameters to be monitored during the study.

To sum up, the basic design for a longevity study with a full survival curve requires ~250 (176 males and 72 females) genetically heterogeneous mice (UM-HET3) per group to detect a 10% lifespan extension with 0.05 type I error and a power of 80% in a two-group design for up to 36-month housing. Drug intervention can be initiated as early as 4 months of age or later in life (16-20 months of age) depending on the cost of compound synthesis and proposed mode of action. Baseline characterization for randomization is recommended to minimize inter-group variation. Test compound prepared in chow diet is widely accepted to avoid introducing a “sham” group if other administration route is adopted, as well as reducing the death caused by extensive laboratory practice. Body weight and body temperature should be monitored during the study as a predictive index for humane endpoint.

## Enhanced Design Options (Budget Permitting)

**Figure 2.**
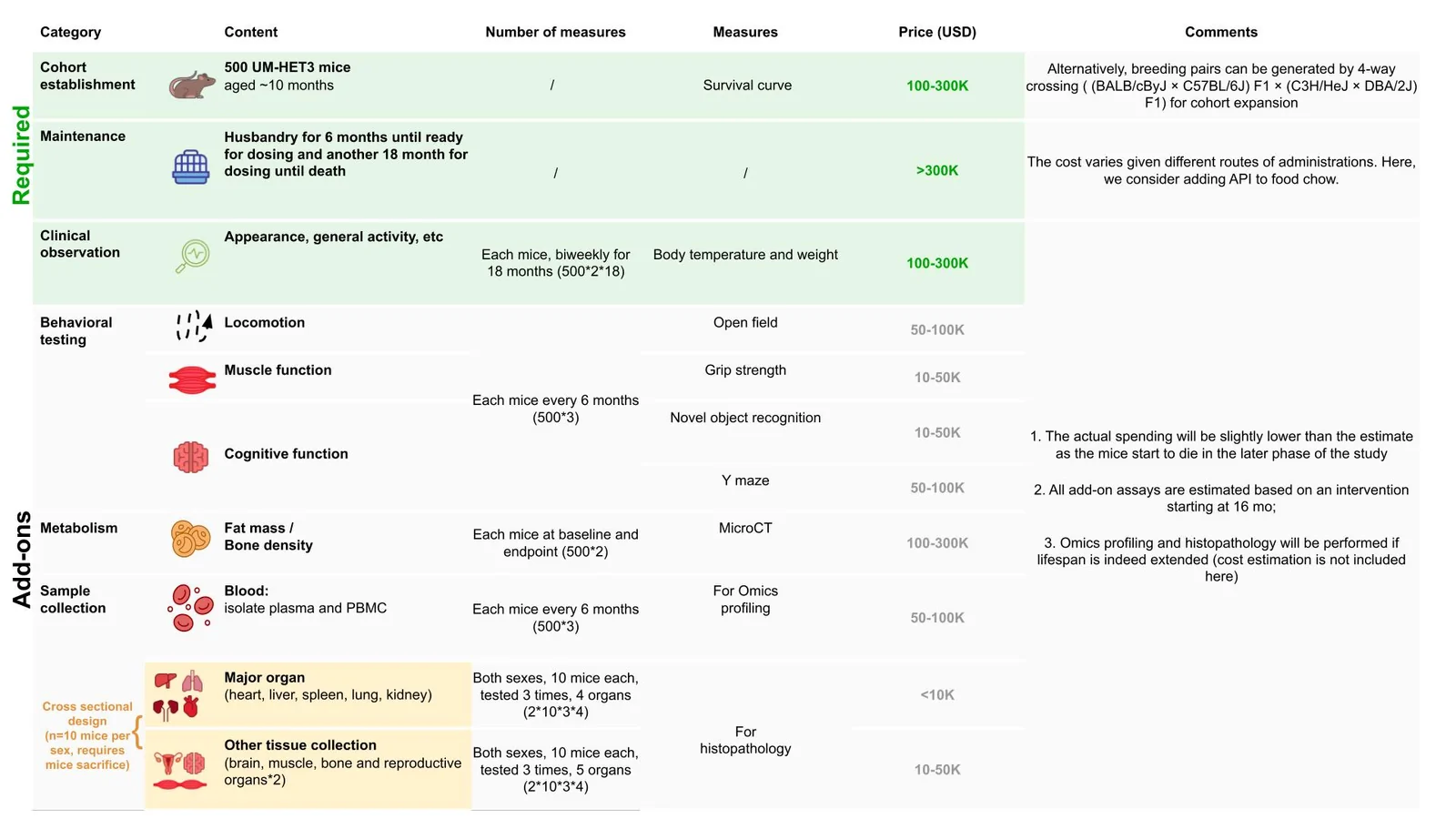
**Estimated costs and detailed description of the required and optional components of the protocol design**. The table outlines the costs of essential and optional components of a mouse lifespan study using UM-HET3 mice. Required elements include animal procurement, maintenance, and biweekly health monitoring. Optional components such as behavioral assays, metabolic assessments, and multi-omic or histopathological analyses can be added to enhance the depth of data.

**Figure 3.**
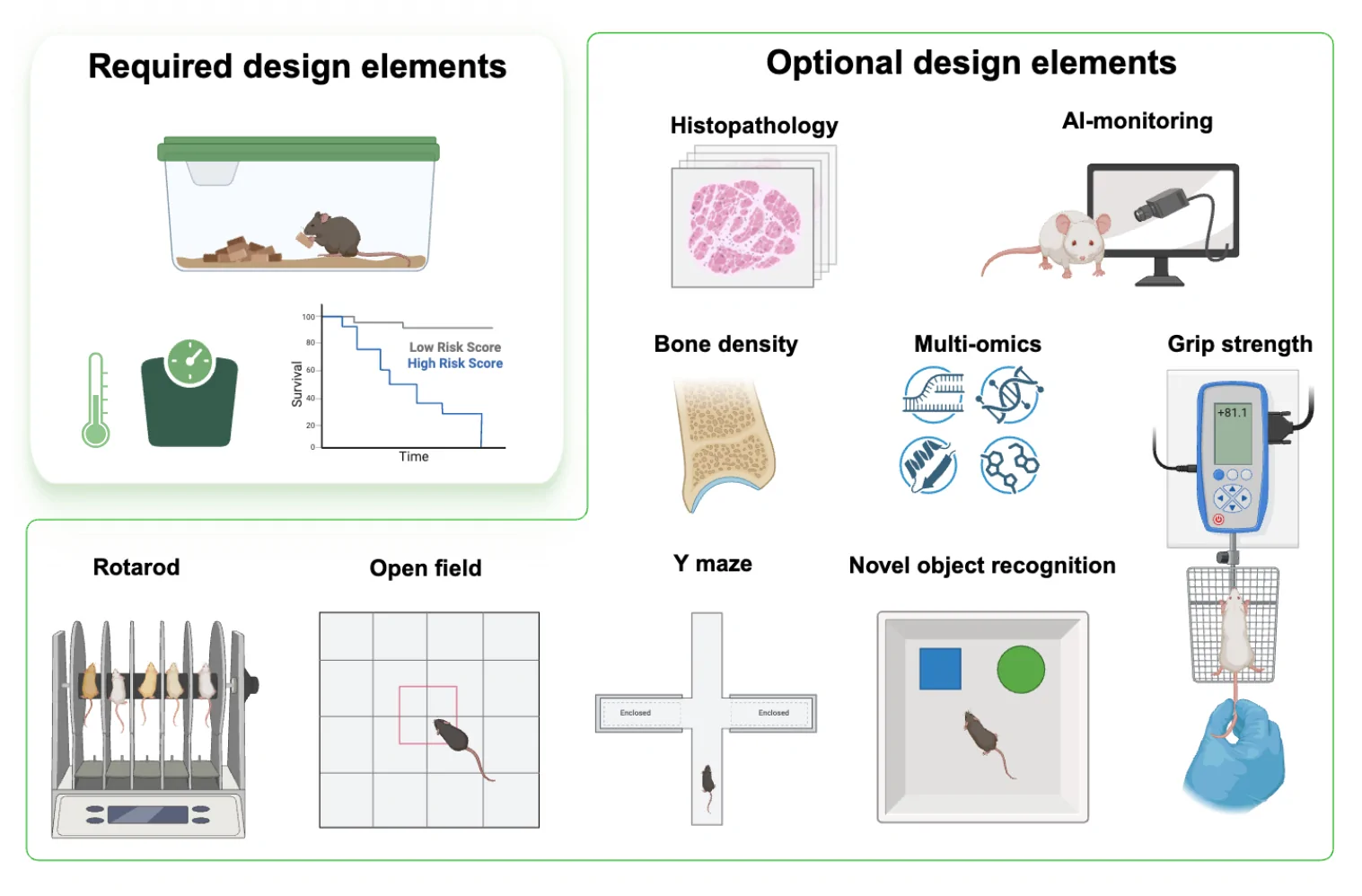
**Visual representations of possible additional tests to be included in the enhanced version of the protocol.** Required elements are placed in the upper left box and include housing and maintenance of mice, temperature and body weight monitoring, and survival tracking via lifespan curve analysis. Optional enhancements placed in the green asymmetrical frame include histopathological evaluation of tissues, AI-based behavioral monitoring, bone density analysis, multi-omics profiling, grip strength testing, and behavioral assays such as the rotarod, open field test, Y maze, and novel object recognition.

If budget permits, additional advanced methods can be incorporated to enhance the study's depth and precision, as outlined in figure 2. These include automated video tracking with AI-based imaging for non-invasive, detailed aging-related data collection, longitudinal assessments of aging phenotypes, periodic blood sampling for multi-omic profiling, and endpoint tissue collection for histological or cross-sectional evaluations. These additions can be bundled by CROs for specific purposes of a drug development program. Figure 2 represents the estimated costs of the study, specifying each required or optional component and the possible times of measurement during the study, showing both optional and required assays. Mice purchase and maintenance are estimated to be the most expensive components. Out of optional assays, multi-omic profiling could be the costliest addition. Finally, figure 3 provides a visual representation of specific tests that could be performed.

### 1. Automatic video tracking combined with AI imaging feature extraction

Intelligent cage systems are now available for basic assessments of the mice, including measuring food intake and water consumption, taking photos of the mice, as well as recording their movement trajectories to reflect their vitality. With AI technology, the digital information collected by the intelligent cages could be used to monitor some of the FI parameters as well as to speculate features associated with longevity with minimal disturbance of mice.

### 2. Longitudinal aging phenotype assessment

Aside from the primary endpoint, which is the lifespan change, other major organ aging phenotypes are also worthy of investigation. For example, changes in body composition, bone density, decreased sperm count/activity or oocyte quality, motor and cognitive impairment, may reflect the aging process in muscle [28], skeleton [29], reproductive organs [30] and brain [31]. Some of these phenotypes could be periodically and non-invasively measured during the intervention by methods such as MRI and various behavioral tests, including but not limited to general open field test, grip strength for muscle functionality, rotarod for movement coordination, Y maze and novel object recognition for cognitive assessment, or core body temperature.

### 3. Periodic plasma, peripheral blood mononuclear cells or blood sample collection for later multi-omic profiling

Biomarkers are critical indicators of aging that are especially useful in lifespan studies since obtaining them does not involve animal sacrifice [32]. They allow researchers to monitor aging progression non-invasively, particularly with the identification of aging clocks based on DNA methylation [33], transcriptomics [34], proteomic panels [35] and metabolomic approaches [36]. The Biomarkers of Aging Consortium has classified biomarkers of aging into 4 major categories: predictive/prognostic, response, surrogate endpoint and discovery, and listed a number of human predictive biomarkers of aging associated with various age-related conditions and their commercial applications [37].

In contrast to biomarkers of aging, which indicate the extent of an individual’s progression along the aging spectrum, aging rate indicators (ARI), conceptualized by Miller et al [38], measure the speed at which age-related changes occur.

Candidate ARIs include physiological changes seen in fat tissue, fat-associated macrophages, muscle, liver, brain, and plasma, as well as molecular measurements reflecting the activity of mTORC1, selective mRNA translation, or each of six MAP kinases in two distinct MAPK cascades in liver, muscle, or kidney. For example, Irisin, a myokine produced by cleavage of muscle protein FNDC5, has been reported to mediate fat tissue metabolism, and its plasma level is significantly elevated in various genetically slow-aging mice and mice whose lifespan is extended by diets and drugs [38]. Another example is glycosylphosphatidylinositol (GPI)-specific phospholipase D1 (GPLD1), a liver-derived GPI-degrading enzyme, whose plasma level was found increased after exercise and correlated with improved cognitive function in aged mice, as well as elevated in active, healthy elderly humans [39].

In addition to biomarkers of aging and ARIs, recent research has identified specific genetic factors that actively modulate the aging process. A number of genes are identified as gerogenes and gerosuppressor genes, whose overactivation and inactivation lead to alteration of the aging process, such as IGF1 and DNMT3A, respectively [40].

Periodic profiling and assessment of these biomarkers of aging and ARIs during the intervention would provide rich information to guide more sophisticated adjustments to the dosing regimen, termination or extension of the intervention and design of assay panels. To minimize the negative impacts of extensive blood drawings on mouse lifespan, we recommend collecting blood samples every 6 months. The blood samples will be stored for profiling when the study meets the primary endpoint.

### 4. Cross-sectional evaluation of testing compounds

The rate of aging differs among organs and may be associated with organ-specific age-related disease and mortality [41]. For aging phenotypes that must be terminally assessed such as major organ histopathology (e.g., Geropathology Grading Platform scoring of heart, liver, kidney, muscle) it may be useful to design a cross-sectional study. For the modified design, major organs could be collected at the endpoint. For the basic design, a separate batch of mice (at least n=10 per sex for vehicle and treatment group, respectively) at each selected time point. Moreover, these phenotypes may involve distinct regulatory pathways which may be specifically targeted by the testing compounds. Therefore, incorporating these specific aging phenotypes parallel to a lifespan study could help to understand the molecular basis of the anti-aging effect of the testing compounds.

## Concluding Remarks

There are multiple benefits for biotech companies to conduct lifespan studies in mice.

1. In parallel to toxicology studies, a lifespan study in mice could provide additional safety information for the testing compounds.
2. A lifespan study in mice could expand the indications of tested compounds and diversify the company’s pipeline, attracting a wider range of potential consumers at all ages who care about healthy longevity.

Aging research may engage communities with shared interests to perform studies in large batches, thus reducing study costs and increasing productivity.

We advocate that sponsors of longevity studies in mice could deposit and share the data resources via an open-access platform to allow systematic investigations on mechanisms of aging. Meta-analysis of historical data could retrospectively shed light on the actual sample size needed, and ideal dosing regimen design for each category of testing compounds and guide new study designs.

We hope this perspective could prompt drug developers to generate high-quality, reproducible and cost-effective results in mice to provide insights into how the drug could eventually affect humans after long term use and hopefully contribute to identifying novel geroprotectors.

## Supplementary Materials

The Supplementary data can be found online at: www.aginganddisease.org/EN/10.14336/AD.2025.0508
