# Supplementary material for: A Standardized Protocol for Mouse Longevity Studies in Preclinical Drug Development
Source: Aging Dis. 2025 Jun 2;17(3):1169–79. doi: 10.14336/AD.2025.0508 (PMC13061545; doi:10.14336/AD.2025.0508)
Supplement: Supplementary file 1 — The Supplementary data can be found online at: www.aginganddisease.org/EN/10.14336/AD.2025.0508 [file AD-17-3-1169-s.pdf]

SUPPLEMENTARY DATA

A Standardized Protocol for Mouse Longevity Studies in Preclinical Drug Development

Alex Zhavoronkov, Qian Wang, Yujie Liu, Wenbin Hou, Yuelei Shen, Dominika Wilczok, Kristen Fortney, Alex Aliper, Man Zhang, Feng Ren, Richard A. Miller

© 2025. Zhavoronkov A et al. Published online at http://www.aginganddisease.org/EN/10.14336/AD.2025.0508

SUPPLEMENTARY DATA

Supplemental Table 1. Statistical Description for Males by Sites

**Site**	**TJL**	**UM**	**UT**
N	1617	1462	1612
Mean	746.56	821.32	735.57
SD	242.69	235.42	240.75

Supplemental Table 2. Statistical Description for Males by Years

**Year**	**2004**	**2005**	**2006**	**2007**	**2009**	**2010**	**2011**	**2012**	**2013**	**2014**	**2015**	**2016**	**2017**	**2018**	**2019**	**2020**	**Total**
N	350	357	309	276	295	274	294	284	273	273	264	275	285	292	297	293	4691
Mean	760.90	771.98	796.56	769.14	785.07	762.61	753.47	774.88	797.75	793.19	743.75	761.60	695.75	706.48	772.75	808.94	766.08
SD	223.09	243.33	210.97	237.97	254.79	250.11	239.05	243.34	239.83	228.22	246.23	229.09	266.62	258.62	249.90	236.58	242.64

Supplemental Table 3. Statistical Description for Females by Sites

**Site**	**TJL**	**UM**	**UT**
N	1457	1450	1489
Mean	884.14	875.00	866.30
SD	177.15	172.24	174.05

Supplemental Table 4. Statistical Description for Females by Years

Year	2004	2005	2006	2007	2009	2010	2011	2012	2013	2014	2015	2016	2017	2018	2019	2020	Total
N	271	289	237	275	242	264	281	279	287	279	274	303	276	286	282	271	4396
Mean	862.07	870.25	863.62	859.95	871.06	891.59	876.74	877.33	867.08	884.35	900.11	873.07	853.76	875.66	881.05	892.87	875.08
SD	177.66	177.39	171.25	191.94	167.90	184.33	167.85	181.21	170.52	189.41	166.24	160.41	168.77	155.61	184.45	173.04	174.61

Supplemental Table 5. Sample Sizes for Males of 2006 (Minimum sample size)

Alpha	%Lifespan extension	Power	N	n1	n2	Mean1	Mean2	SD
0.05	10	0.8	224	112	112	796.56	876.22	210.97
0.05	15	0.8	100	50	50	796.56	916.04	210.97
0.05	20	0.8	58	29	29	796.56	955.87	210.97

Supplemental Table 6. Sample Sizes for Males of 2017 (Maximum sample size)

Alpha	%Lifespan extension	Power	N	n1	n2	Mean1	Mean2	SD
0.05	10	0.8	464	232	232	695.75	765.33	266.62
0.05	15	0.8	208	104	104	695.75	800.11	266.62
0.05	20	0.8	118	59	59	695.75	834.90	266.62

Supplemental Table 7. Sample Sizes for Males of TJL

Alpha	%Lifespan extension	Power	N	n1	n2	Mean1	Mean2	SD
0.05	10	0.8	334	167	167	746.56	821.22	242.69
0.05	15	0.8	150	75	75	746.56	858.54	242.69
0.05	20	0.8	86	43	43	746.56	895.87	242.69

© 2025. Zhavoronkov A et al. Published online at http://www.aginganddisease.org/EN/10.14336/AD.2025.0508

SUPPLEMENTARY DATA

Supplemental Table 8. Sample Sizes for Males of UM

Alpha	%Lifespan extension	Power	N	n1	n2	Mean1	Mean2	SD
0.05	10	0.8	260	130	130	821.32	903.45	235.42
0.05	15	0.8	118	59	59	821.32	944.52	235.42
0.05	20	0.8	68	34	34	821.32	985.58	235.42

Supplemental Table 9. Sample Sizes for Males of UT

Alpha	%Lifespan extension	Power	N	n1	n2	Mean1	Mean2	SD
0.05	10	0.8	340	170	170	735.57	809.13	240.75
0.05	15	0.8	152	76	76	735.57	845.91	240.75
0.05	20	0.8	88	44	44	735.57	882.68	240.75

Supplemental Table 10. Sample Sizes for Males by Years (Lifespan increased by 10%)

Yea r	200 4	200 5	200 6	200 7	200 9	201 0	201 1	201 2	201 3	201 4	201 5	201 6	201 7	201 8	201 9	202 0	Tot al
N	272	314	224	304	334	340	318	312	286	262	346	288	464	424	332	272	318

Supplemental Table 11. Sample Sizes for Females of 2018 (Minimum sample size)

Alpha	% Lifespan extension	Power	N	n1	n2	Mean1	Mean2	SD
0.05	10	0.8	102	51	51	875.66	963.23	155.61
0.05	15	0.8	48	24	24	875.66	1007.01	155.61
0.05	20	0.8	28	14	14	875.66	1050.79	155.61

Supplemental Table 12. Sample Sizes for Females of 2007 (Maximum sample size)

Alpha	%Lifespan extension	Power	N	n1	n2	Mean1	Mean2	SD
0.05	10	0.8	160	80	80	859.95	945.95	191.94
0.05	15	0.8	72	36	36	859.95	988.94	191.94
0.05	20	0.8	42	21	21	859.95	1031.94	191.94

Supplemental Table 13. Sample Sizes for Females of TJL

Alpha	%Lifespan extension	Power	N	n1	n2	Mean1	Mean2	SD
0.05	10	0.8	130	65	65	884.14	972.55	177.15
0.05	15	0.8	60	30	30	884.14	1016.76	177.15
0.05	20	0.8	34	17	17	884.14	1060.97	177.15

Supplemental Table 14. Sample Sizes for Females of UM

Alpha	%Lifespan extension	Power	N	n1	n2	Mean1	Mean2	SD
0.05	10	0.8	124	62	62	875.00	962.50	172.24
0.05	15	0.8	58	29	29	875.00	1006.25	172.24
0.05	20	0.8	34	17	17	875.00	1050.00	172.24

© 2025. Zhavoronkov A et al. Published online at http://www.aginganddisease.org/EN/10.14336/AD.2025.0508

SUPPLEMENTARY DATA

Supplemental Table 15. Sample Sizes for Females of UT

Alpha	%Lifespan extension	Power	N	n1	n2	Mean1	Mean2	SD
0.05	10	0.8	130	65	65	866.30	952.93	174.05
0.05	15	0.8	60	30	30	866.30	996.25	174.05
0.05	20	0.8	34	17	17	866.30	1039.56	174.05

Supplemental Table 16. Sample Sizes for females by Years (Lifespan increased by 10%)

Year	2004	2005	2006	2007	2009	2010	2011	2012	2013	2014	2015	2016	2017	2018	2019	2020	Total
N	136	134	126	160	120	138	118	136	124	146	110	108	126	102	140	120	128

© 2025. Zhavoronkov A et al. Published online at http://www.aginganddisease.org/EN/10.14336/AD.2025.0508
